# Stephen's Hospital, Dublin

**Published:** 1832-05

**Authors:** 


### STEPHEN'S HOSPITAL, DUBLIN.

*Clinical Remarks, by* Mr. Cusack, *on a Case of Circumscribed Brachial Aneurism, cured by Compression and Digitalis.**

Case. J. M., æt. twenty-six, a countryman, was admitted into this hospital on the 11th of October, under the care of Mr. Cusack, for circumscribed brachial aneurism at the bend of the right arm.

He states, that on the 29th of last month, being unwell, he applied to a "bleeder" in the country, for the purpose of having some blood taken from the arm. Nothing remarkable occurred during the operation, except that the blood was propelled to a much greater distance (thirteen feet) than usual from the wound. On the next morning the entire of the palmar surface of the upper extremity, from the wrist to the axilla, was swollen, and of a purple colour. The bandage applied by the "bleeder" feeling tight, he caused it to be loosened, and directed the patient to keep the limb

* The Lancet.

wet with vinegar and water. A week subsequent to this period, the swelling being much diminished, and the purple colour being in some parts changed to a yellow hue, he observed a small beating tumor immediately beneath the wound in the integuments. As this remained stationary, he applied, after some days, to a neighbouring surgeon, who at once detected the nature of the accident, and sent him up to this hospital. There is now (11th of October) a firm pulsating tumor, about the size of a pigeon's egg, in the site of the wound made by the lancet. Pressure on the brachial artery in the middle of the arm completely stops pulsation in the aneurism, and reduces it in size; there is considerable ecchymosis throughout the arm generally; the wound has not healed, and the skin round it appears thinned. The pulsations of the radial artery on this side are not near so strong as in the opposite arm.

A roller was applied, with a moderate degree of pressure, from the fingers up to the axilla, a compress of lint having been previously laid upon the tumor. He was desired to keep the limb around the tumor constantly wet with cold water. A state of perfect quietude was enjoined, and the patient was put on low diet.

15. The extravasated blood is almost removed; the tumor remains unaltered; roller reapplied; cold water to be continued, and the state of rest, and low diet, to be strictly observed.

17. Tumor firmer than at last report; the remainder of the limb has regained its natural appearance; the integuments round the wound are more firm. Ordered to have twenty ounces of blood extracted from the opposite arm, and to take ten drops of the tincture of digitalis three times a day; low diet and cold water to be continued; bandage reapplied.

18. Pulse less frequent than yesterday; continue the digitalis, cold water, and low diet.

20. Pulsation in the tumor diminishing; it feels more firm; and does not subside when pressure is made on the brachial artery; wound healed; pulse seventy; bandage reapplied, a piece of sponge being substituted for the lint. Continue remedies.

22. Pulsation in the tumor very indistinct; the tumor itself is firmer and smaller than at last report; pulse sixty-six; sponge and roller reapplied; tincture of digitalis increased to twenty drops three times a day; cold water and low diet to be continued.

28. Pulsation in the tumor nearly gone; the swelling is diminishing very rapidly; pulse at the wrist more strong than at last report; roller and compress reapplied; dose of tincture increased to gtt. xx., secundâ quâque horâ.

Ten P.M. Has taken six doses of the tincture since morning; pulse forty, and weak; not much giddiness of the head, but scintillæ are flying before the eyes. No sickness of stomach.

30. No pulsation in the tumor; the skin over it quite sound, and not adherent to the swelling as it was at first. Bandage and compress reapplied; digitalis to be continued.

November 2d. Bandage reapplied yesterday; complains of its being tight; digitalis to be continued.

3. Tumor much diminished; the arm has regained its natural appearance; the pulse at the wrist strong and natural; digitalis to be omitted; roller reapplied.

21. From the time of the last report the tumor gradually subsided; there is now no trace of it. Dismissed the hospital cured.

*Clinical observations.* In performing the operation of venesection at the bend of the arm, there are a variety of ill consequences which will now and then ensue, even in the hands of the most skilful surgeon. The chief of these are, "inflammation of the vein," a "wound of the nerves," which in delicate females in good society is sometimes followed by the most distressing symptoms, indicative of great nervous irritation by "inflammation of the semilunar fascia of the biceps;" but the most important is the "wounding of the brachial artery," which latter circumstance may occur in one of the two following ways: 1st. By the vein and artery being transfixed by the same thrust of the lancet; or, 2dly, by the vein rolling on one side, while the lancet passes through the integuments, and wounds the vessel without touching the vein. When the artery and vein are both wounded, a tumor sometimes forms between them, which is a diverticulum to the blood flowing in the artery, accompanied by a thrilling and hissing noise; this constitutes that form of aneurism which has been denominated "aneurismal varix." Or we may have no tumor formed between the artery and vein, but adhesion takes place between their coats at the points of the corresponding wounds, while the anterior wound in the vein corresponding to that in the integuments heals; there is thus a direct communication established between the artery and vein, which gives rise to that kind of aneurism which authors call "varicose aneurism." When the artery of the arm has been wounded directly, a "circumscribed aneurism" may be the consequence, as in the case we are about to consider; or the blood may be diffused extensively throughout the cellular membrane, causing enormous distention, which is generally greater above than below the elbow: this disease is the "diffused aneurism" of surgical writers.

In all cases of the kind which we are now considering, our attention should be directed to the external hemorrhage, which we must repress; sometimes both to the external and internal hemorrhage, that we may prevent the blood from becoming diffused throughout the cellular membrane. I shall on the present occasion confine myself to the history and symptoms of the present case, but I thought it right to premise so far, in order that I might be better understood by the junior members of the class.

The "bleeder," as he is called in the country, was aware of the nature of the injury he had inflicted, and of the consequences which would ensue; he therefore immediately applied pressure to the part, in order to arrest the hemorrhage; yet the bleeding went on internally in a slight degree. Why was this extravasation slight, and why was not the blood diffused to a greater extent throughout the limb? The reason is, that the pressure employed formed a cyst of the cellular membrane, which was circumscribed by the adhesive inflammation, and gave to the tumor as firm a pulsation as an artery. This is the least formidable kind of injury occurring after the operation of venesection.

In bleeding, a great deal of caution is necessary, and although we may cry out against a person who has the misfortune to wound the artery, yet we find the same accident happen in the hands of good surgeons. It remains for us to consider how far the treatment adopted in the present case has proved beneficial, how far the consequences have been satisfactory, and also to point out the nature of the disease, and the differences between it and a common encysted tumor.

We are not to imagine that every tumor which conveys to the touch the sensation of pulsation is an aneurism. How then would a surgeon know whether this was or was not a case of aneurism? By simply attending to the history of the case, and the nature of the symptoms above detailed; fluctuation was distinct; pressure on the artery above the tumor diminished it in size, and put a stop to all pulsation; by removing the pressure, the tumor returned to its original dimensions, and pulsation again became distinct; but if it were an encysted tumor, we should have had none of these symptoms. If you examine the state of the pulse at the wrist in the affected side, you will find it considerably weaker than in the opposite wrist, because the blood is diverted from its natural course into the aneurismal sac. This is not the case in encysted tumors. By raising them up with the fingers, we do away with all pulsation, which is a tremulous motion rather than an actual pulsation, but we do not affect the original size of the tumor; it is therefore necessary to make a careful survey of the symptoms, and of the history of the case, that we may be enabled to form a just and accurate diagnosis.

*Treatment.* What mode of treatment would you adopt in a case of circumscribed aneurism? We find that a roller and compress, with everything which tends to diminish the impetus of the circulation, to obliterate the sac, and to prevent inflammation, are the best remedies we can adopt. In applying the bandage, commence at the fingers; for if you neglect this, the hand will become swollen and œdematous, and you had better bandage each of the fingers separately, then include them in the roller which you intend to carry up the arm, and when you come to the bend of the elbow, place a piece of sponge on the tumor; let the pressure be light at first, if not, you are apt to excite inflammation. After this has been done, place dossils of lint in the line of the artery leading to the tumor, for the purpose of diminishing the impetus of the blood which flows through the artery and feeds the aneurismal sac.

The constitutional treatment will have no effect, unless you attend particularly to these circumstances. We are now to determine what this constitutional treatment should be. Perfect rest. Bleed the patient in the opposite arm, if repeated hemorrhages have not taken place previously; for it often happens that hemorrhage will occur ten or fifteen times, when the orifice of the wound has not closed; in such case the pulse will be 120, there will be great thirst, &c., and even though the pulse be hard and jarring, yet it would be improper to bleed him while labouring under this kind of fever, which has been called the hemorrhagic. You should therefore bleed the patient, if the above circumstances do not contra-indicate it; confine him to a low diet, giving such food as will not increase the action of the heart and arteries, and which does not contain much nutriment. You should also administer the tincture of digitalis, if there be no organic disease; it reduces the frequency of the pulse, and is a valuable remedy when the heart or lungs are not diseased. In this man it produced the desired effect, for we find that when first administered, his pulse was as high as 112; but when he was brought under the effects of the digitalis, his pulse was reduced to forty, to that quiet state most favorable for the accomplishment of a cure: accordingly we find a manifest change takes place in the tumor, the blood becomes more firm in it, the tumor begins to subside, the pulse at the wrist becomes more obvious, and of its original standard. These latter effects do not take place, until the tumor becomes strong and firm, because, previous to this change, the blood continues to flow into the aneurismal sac, but the recovery of the pulse shews it begins to take its natural course.

It is a remarkable thing that he did not complain of giddiness in the head, or sickness of the stomach, but merely of scintillæ flying before his eyes: these are usual symptoms, and the surgeon commonly asks his patient if he feels any of them; after which lowering of the pulse ensues, which always proves successful, as it did in the present case. But we are to observe, that every case of this disease is not one fit for the above treatment. For example, if there be any appearance of inflammation in the tumor, the requisite pressure would only cause pain, and it would be folly to use it until the pain was reduced, and all inflammatory action in the tumor removed. You see, therefore, if you employ pressure in such cases as we have supposed, you would only aggravate the symptoms, and set aside all possibility of accomplishing a cure; it is therefore necessary to remove all pain, œdema, &c., consequent on the inflammation, before the means of radical cure are applied, otherwise the disease will go on from bad to worse, and you will have to regret the impropriety of your treatment when it is too late. I do not recollect a single case of circumscribed aneurism, which did not yield to the treatment I have described, and, although an unpleasant disease, it is not so formidable as might, at first sight, appear.

